# Identification and validation of transferrin receptor protein 1 for predicting prognosis and immune infiltration in lower grade glioma

**DOI:** 10.3389/fnmol.2022.972308

**Published:** 2022-11-22

**Authors:** Hongrong Wu, Haoyang He, Jiexiong Huang, Chuan Wang, Yuejiao Dong, Ruilin Lin, Zhuofeng Cheng, Qiancheng Qiu, LiangLi Hong

**Affiliations:** ^1^Department of Pathology, The First Affiliated Hospital of Shantou University Medical College, Shantou, Guangdong, China; ^2^The First Clinical Medical College of Guangdong Medical University, Zhanjiang, Guangdong, China

**Keywords:** TFRC, LGG, prognosis, tumor microenvironment, ferroptosis

## Abstract

**Introduction:**

Transferrin receptor protein 1 (TFRC), an ananda molecule associated with ferroptosis, has been identified as affecting a wide spectrum of pathological processes in various cancers, but the prognostic value correlates with the tumor microenvironment of TFRC in lower-grade glioma (LGG) is still unclear.

**Materials and methods:**

Clinical pathological information and gene expression data of patients with LGG come from The Cancer Genome Atlas (TCGA), Chinese Glioma Genome Atlas (CGGA), GTEx, Oncomine, UCSC Xena, and GEO databases. We then used various bioinformatics methods and mathematical models to analyze those data, aiming to investigate the clinical significance of TFRC in LGG and illustrate its association with tumor immunity. In addition, the molecular function and mechanisms of TFRC were revealed by gene ontology (GO), Kyoto Encyclopedia of Genes and Genomes (KEGG), and gene set enrichment analysis (GSEA). Immunohistochemical experiments and single-cell analysis have been performed.

**Results:**

TFRC expression was highly expressed in many tumors and showed a poor prognosis. Including gliomas, it was significantly associated with several poor clinical prognostic variables, tumor immune microenvironment, tumor mutational burden (TMB), m6a modification, and ferroptosis in LGG. TFRC as a key factor was further used to build a prediction nomogram. The C-index, calibration curve, and decision curve analysis showed the nomogram was clinically useful and calibration was accurate. At the same time, we also demonstrated that promoter hypomethylation of DNA upstream of TFRC could lead to high TFRC expression and poor overall survival. There is a significant correlation between TFRC and CD8 + T cell, macrophage cell infiltration, and several immune checkpoints, such as PD-L1(cd274), CTLA4, and PD1, suggesting a novel direction for future clinical application. Functional and molecular mechanism analysis showed an association of TFRC expression with immune-related pathways through GSEA, GO, and KEGG analysis. Finally, immunohistochemical experiments and single-cell analysis confirmed the expression of TFRC in glioma.

**Conclusion:**

TFRC may be a potential prognostic biomarker and an immunotherapeutic target for glioma.

## Introduction

Glioma is one of the most common malignant tumor types in the brain or spine tumors formed by glial cells. Compared with glioblastoma (GBM), lower-grade glioma (LGG) grows slowly with not so much malignancy. However, some LGG develops rapidly into GBM with a poor prognosis (Jakola et al., 2017).

Ferroptosis is a newly described form of regulated cell death; ferroptosis stimulation inhibits tumor growth, improves patient survival, and increases the efficacy of radiation and chemotherapy in the central nervous system neoplasms (Mitre et al., 2022). The transferrin receptor protein 1 (TFRC) gene encodes transferrin receptor 1 (TfR1) or cluster of differentiation 71 (CD71), a human protein. TfR1 is a receptor needed for iron delivery from transferrin to target cells (Fillebeen et al., 2019). However, the role of TFRC in LGG has not been illustrated.

Some reports suggest that ferroptosis is associated with the tumor microenvironment and posttranscriptional regulation (Demuynck et al., 2021; Shen et al., 2021). Some studies have shown that tumor-infiltrating lymphocytes play different vital roles in tumor progression by ferroptosis-related molecular mechanisms (Zhou and Lu, 2017). Nevertheless, the effect of TFRC on the microenvironment in LGG has not been described.

Therefore, we illustrated the association between TFRC expression and the tumor prognosis, clinical–pathological parameters, immunity, tumor mutational burden (TMB), and m6A modification in RNA. Our research suggested that TFRC expression may act as a potential effective prognostic marker and target for immunotherapy.

## Materials and methods

### UCSC Xena, GTEx, CGGA Oncomine database, and single-cell analysis

The UCSC Xena database^1^ combines about 200 public databases, including TCGA, TARGET, GTEx, CCL, and ICGC (Goldman et al., 2020). The database is useful for examining gene expression methylation and copy number, gene expression, somatic mutation, and clinical information. We can use the data from the GTEx database to create a normal group for comparison with the tumor group in TCGA using a boxplot (Kruppa and Jung, 2017). In addition, glioma with different subtypes and new types were obtained from the Chinese Glioma Genome Atlas (CGGA)^2^ (dataset ID: mRNAseq_325).

The Oncomine database^3^ integrates GEO databases (Rhodes et al., 2004). This database is primarily used for gene expression analysis. We used the Oncomine database to analyze TFRC expression in normal and tumor tissues.

For a single-cell dataset, TISCH2 provides meta information, cell-type annotation, expression visualization, and differential gene expression (Sun et al., 2021).

### Clinical specimen collection

The tumor tissue specimens of patients with glioma were collected from the pathology department at The First Affiliated Hospital of Shantou University Medical College (Shantou, China). We named it the Shantou cohort. To understand the expression and distribution of TFRC in tumor tissues, we collected 10 specimens of low-grade gliomas and 10 specimens of high-grade gliomas. Hematoxylin-eosin staining of the tissues was confirmed by two different pathologists. This study was approved by the Institutional Review Board of The First Affiliated Hospital of Shantou University Medical College, and informed consent was obtained from all individuals.

### Cox regression analysis and nomogram for predicting survival

The comparison of OS, DSS, PFI, and DFI between two groups was performed by the Kaplan–Meier method. Both univariate Cox regression and multivariate Cox regression analyses were performed to assess the prognostic significance of TFRC in patients with LGG, and they were visualized using the R package “forest” (Clark et al., 2001). A nomogram is a graphical representation of these factors that can be used to predict the risk of recurrence by the points correlated to each risk factor through the R package “rms.” The indexes of ROC, calibration curves, and DCA, were used for the discrimination, calibration, and clinical practicability of this model.

### Gene expression profiling and DNA methylation analyses

The UCSC Xena platform includes gene expression and DNA methylation data, and it was designed for visualization and data analysis (Goldman et al., 2020). MethSurv is a web measurement for performing survival analysis through methylation data (Modhukur et al., 2018). We applied these two bioinformatics tools for correlation analysis between gene expression and DNA methylation. The ggpairs function of the R package GGally was used to visualize the correlation for each pair of variables.

### Tumor microenvironment

The tumor microenvironment comprises infiltrating immune cells, stromal cells, tumor cells, and an extracellular matrix. Furthermore, the tumor microenvironment of LGG consists primarily of non-tumor cells, such as stromal cells and immune cells (Deng et al., 2020; Guo et al., 2020). The ESTIMATE algorithm can be used to assess the stromal and immune cell content in tumor tissue, predict the immune and stromal scores, and calculate the tumor purity in each tumor sample (Sturm et al., 2019; Meng et al., 2020). The ICB response map was made using the TIDE algorithm with the R packages “ggplot2” and “ggpubr” (Jiang et al., 2018).

### Differential genes expression, gene ontology, and Kyoto Encyclopedia of Genes and Genomes analysis

In the LGG-TCGA databases, we first grouped the patients according to median values of TFRC mRNA value, if it was greater than the median value; it was defined as the high expression group. Differentially expressed genes were searched using the Limma package in R software based on the threshold (adjusted *P* < 0.05 and Log2 (Fold Change) > 1 or Log2(Fold Change) <−1). The ClusterProfiler package in R was used to perform GO functional analysis of potential targets and enrich KEGG pathways (Yu et al., 2012).

### Immunohistochemistry

In the Shantou cohort, the tissue sections were incubated with boiled citrate buffer for 15 min and 3% hydrogen peroxide for 10 min. Anti-transferrin receptor (ab214039) antibody and anti-PD-L1 (ab205921) were purchased from Abcam (Cambridge, UK). The sections were incubated at 4°C overnight with the primary antibodies anti-TFRC (Abcam, ab84036, 1:300). The intensity of positive cells was verified by two separate pathologists. The Image-Pro Plus 7.0 software was used to assess the percentage of positive cells.

Moreover, formalin-fixed paraffin-embedded sample specimens of patients with glioma were obtained from the Human Protein Atlas Project.^4^ This project includes immunohistochemical and cytological experiment data. Immunohistochemistry staining was re-evaluated independently by two experienced pathologists. The quantification of immunohistochemistry (IHC) staining of TFRC and PD-L1 was performed by Image-Pro Plus 7.0. The combined positive score (CPS) is defined as the number of positive tumor cells, lymphocytes, and macrophages, divided by the total number of viable tumor cells multiplied by 100 (de Ruiter et al., 2021).

### Statistical analysis

The statistical analysis diagrams shown above were produced by the R software (4.1.0).

## Results

### Upregulation of transferrin receptor protein 1 in multiple tumors including lower-grade glioma

**FIGURE 1:**
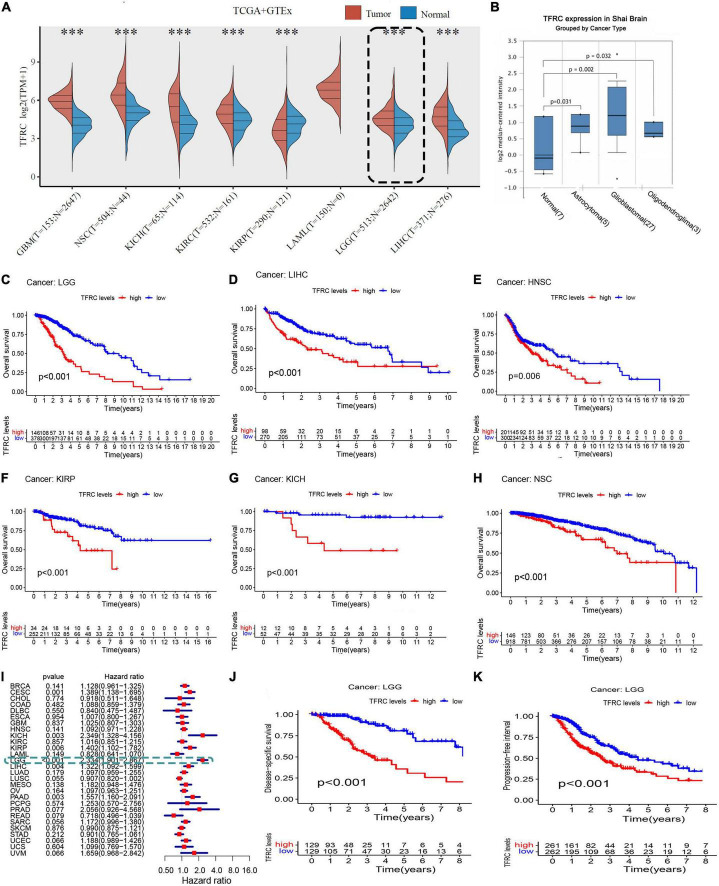
Association of TFRC expression level and survival in LGG. **(A)** TFRC mRNA expression in TCGA LGG tissues and GTEx adjacent normal tissues. **(B)** TFRC expression in GEO. **(C–H)** TFRC expression effect on OS in TCGA tumor patients by Kaplan–Meier survival analysis. **(I)** Forest plot showing TFRC expression effect on OS in TCGA tumor patients by Cox regression. Association of TFRC expression level and survival in LGG. **(J)** TFRC mRNA expression correlation with DSS. **(K)** TFRC mRNA expression correlation with PFI. ***p < 0.001.

A schematic diagram of this study is displayed in Supplementary Figure 1. Through a comparative analysis of the expression data from the GTEx (dbGaP Accession phs000424.v8. p2) and TCGA databases, we found that TFRC had a higher expression in multiple cancers, including LGG, glioblastoma multiforme (GBM), invasive breast carcinoma no special type (NST), kidney chromophobe cancer (KICH), kidney renal clear cell carcinoma (KIRC), kidney renal papillary cell carcinoma (KIRP), acute myeloid leukemia (AML), and liver hepatocellular carcinoma (LIHC), than in the corresponding normal tissues (Figure 1A and Supplementary Figure 2). To verify the expression pattern of TFRC in LGG, we analyzed the Oncomine database and found that there was also a higher TFRC expression level in different kinds of glioma than in the normal group (Figure 1B).

### High transferrin receptor protein 1 expression causes low survival

In the TCGA database, higher TFRC expression was significantly associated with poorer overall survival (OS) (Figure 1C, *P* < 0.00), disease-specific survival, and progression-free interval (Figures 1J,K, *P* < 0.001) in LGG. On the other hand, TFRC expression has a significant effect on OS in patients with LIHC, HNSC, KIRP, KICH, and NSC (Figures 1C–H, *P* < 0.05). Furthermore, the forest plot of covariates in the Cox proportional hazards model indicated that the *P*-values less than 0.05 (<0.05) are considered to be statistically significant in LGG, CESC, HNSC, KIRC, and LUSC and that the hazard ratio of LGG was 1.269 (1.90–2.87) (Figure 1I). Therefore, those results showed that patients with LGG and high TFRC expression levels have a poor prognosis.

### Transferrin receptor protein 1 expression effect on the clinical-pathological parameters in lower-grade glioma

**FIGURE 2:**
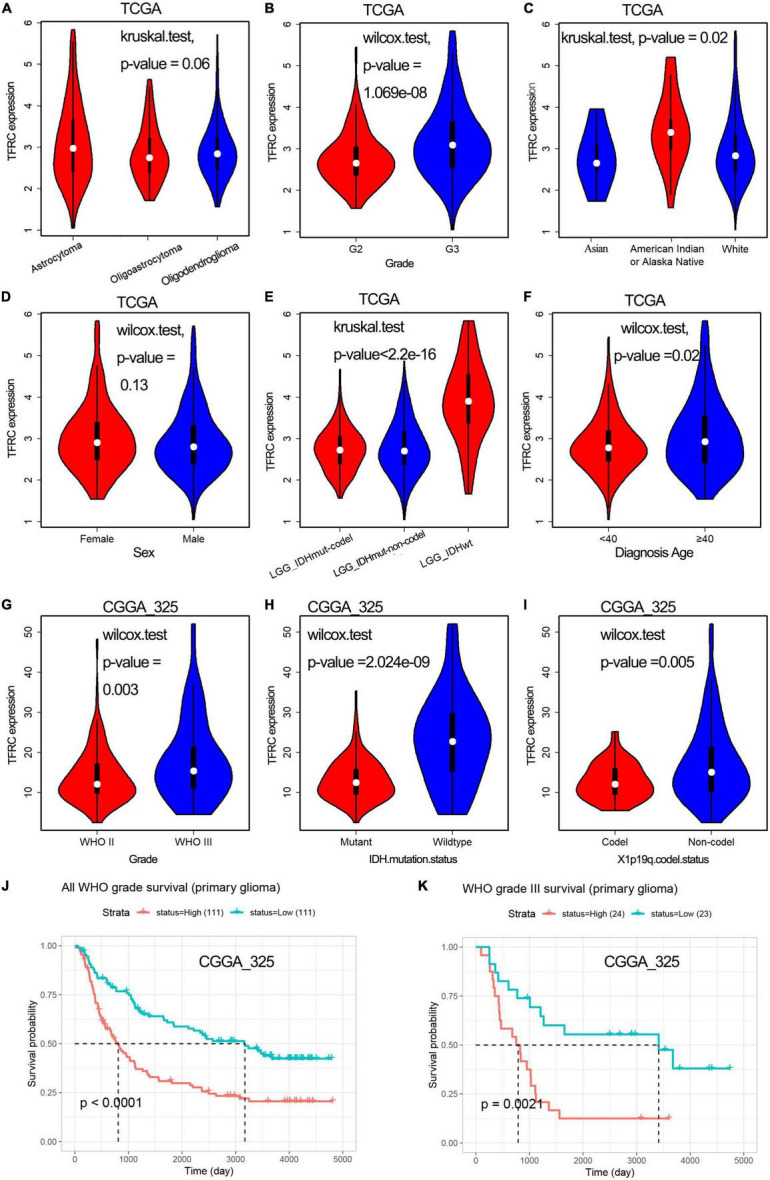
Relationship between TFRC expression and clinicopathologic variables of patients with LGG and OS analysis. **(A–F)** Histopathologic classification **(A)**, histology grade **(B)**, race **(C)**, sex **(D)**, IDH mutation status **(E)**, and diagnosis age **(F)** in TCGA. **(G–I)** Histology grade **(G)**, IDH mutation status **(H)**, chromosomal 1p/19q codeletions **(I)** in CGGA. **(J,K)** Association with TFRC expression and overall survival time.

A total of 499 LGG samples with TFRC expression data and the patients’ clinical-pathological parameters from the TCGA database were analyzed. We used the Chi-square and Fisher-square tests to explore the association (Zhou et al., 2020). Then, we found that TFRC expression was highly correlated with gender (*P* = 0.04326), race (*P* = 0.01443), and histology grade (*P* = 2.11e–06), whereas TFRC expression did not have a close relationship between histological type by Chi-square (Table 1). For histology grade, TFRC expression was significantly higher in G3 than in the others (Figure 2B, *P* < 0.00). For the human population, Black or African Americans had a higher TFRC expression than Whites (Figure 2C, *P* < 0.01). More importantly, for the classification of tumors of the central nervous system (WHO CNS5), patients with isocitrate dehydrogenase (IDH) wildtype have a higher expression level of TFRC than those with isocitrate dehydrogenase (IDH) mutations and chromosomal 1p/19q codeletions (Figure 2E, *P* < 0.00). After reviewing other reports, we took the age of 40 as the boundary and found that people with age ≥ 40 years old had a higher TFRC expression than those with age <40 years (Figure 2F, *P* < 0.05). Nevertheless, the gender and histopathologic classification of our patients did not show a clear difference (*P* = 0.06; Figures 2A,D).

**TABLE 1:** The association with TFRC and clinicopathological characteristics in TCGA-LGG patients.

Variable	N	High, *N* = 249	Low, *N* = 249	*p*-value^b^
**Diagnosis age^a^**	497	43 (33, 57)	39 (32, 50)	0.002
**Cancer type**	498			0.3
Astrocytoma		101 (41%)	87 (35%)	
Low-grade glioma (NOS)		0 (0%)	1 (0.4%)	
Oligoastrocytoma		56 (22%)	69 (28%)	
Oligodendroglioma		92 (37%)	92 (37%)	
**Neoplasm histologic grade**	496			< 0.001
G2		93 (38%)	147 (59%)	
G3		155 (62%)	101 (41%)	
**Sex**	497			0.2
Female		118 (47%)	103 (42%)	
Male		131 (53%)	145 (58%)	
**Subtype**	491			< 0.001
LGG_IDHmut-codel		69 (28%)	94 (38%)	
LGG_IDHmut-non-codel		96 (40%)	142 (57%)	
LGG_IDHwt		78 (32%)	12 (4.8%)	
**Tumor type**	498			0.3
Astrocytoma		101 (41%)	87 (35%)	
Low-grade glioma		0 (0%)	1 (0.4%)	
Oligoastrocytoma		56 (22%)	69 (28%)	
Oligodendroglioma		92 (37%)	92 (37%)	

^a^Median (IQR); *n* (%). ^b^Wilcoxon rank-sum test; Fisher’s exact test; Pearson’s Chi-squared test.

Furthermore, in the CGGA database, the Table 2 and Figures 2G–I showed that the expression levels of TFRC were also correlated with the histology grade (*P* = 0.003), IDH mutation status (*P* < 0.001), and chromosomal 1p/19q codeletions (*P* = 0.005). Meanwhile, patients in the high TFRC expression group had a poorer survival time than those in the low TFRC expression group, especially in WHO Grade III (Figures 2J,K).

**TABLE 2:** The association with TFRC and clinicopathological characteristics in CGGA-LGG patients.

Variable	*N*	High, *N* = 92	Low, *N* = 93	*p*-value^b^
**PRS.type**	181			0.9
Primary		70 (80%)	73 (78%)	
Recurrent		18 (20%)	20 (22%)	
**Grade**	181			0.002
WHO II		40 (45%)	63 (68%)	
WHO III		48 (55%)	30 (32%)	
**Gender**	185			> 0.9
Female		35 (38%)	36 (39%)	
Male		57 (62%)	57 (61%)	
**Age^a^**	185	41 (35, 47)	38 (34, 44)	0.2
**IDH.mutation.status**	184			<0.001
Mutant		53 (58%)	81 (88%)	
Wildtype		39 (42%)	11 (12%)	
**X1p19q.codel. status**	180			0.015
Codel		22 (25%)	38 (42%)	
Non-codel		67 (75%)	53 (58%)	

^a^ *n* (%); Median (IQR). ^b^Pearson’s Chi-squared test; Wilcoxon rank-sum test.

### Multivariate Cox regression analysis and nomogram model

**FIGURE 3:**
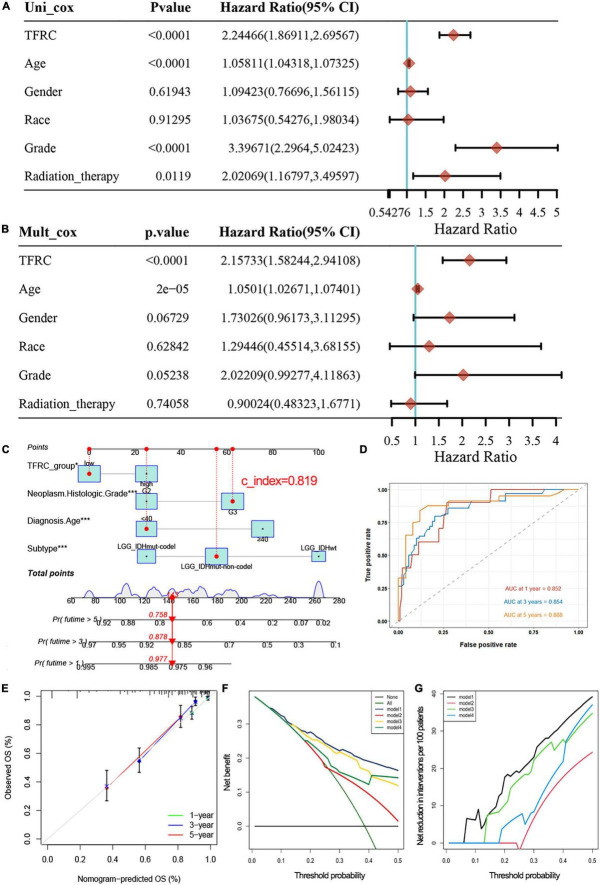
Univariate and multivariate Cox regression analysis in LGG and nomogram predicting. **(A)** Univariate Cox regression. **(B)** Multivariate Cox regression. **(C)** Nomogram predicting probability in LGG. TFRC and 3 clinicopathologic factors are located on each variable axis. The sum of these numbers is located on the Total Points axis, and the three lines are drawn downward to the 1, 3, 5-year survival probability of LGG axes to determine the probability. **(D,E)** The ROC curve showed the area under curve (AUC) values and the nomogram prediction calibration curve. **(F,G)** Decision curve analyses demonstrating the benefit for predicting survival, model 1: TFRC group, Diagnosis Age, Histologic Grade, and Subtype. Model 2: TFRC group. Model 3: TFRC group, Histologic Grade, Subtype. Model 4: TFRC group, Subtype. *p < 0.05, **p < 0.01, and ***p < 0.001.

The expression of TFRC (*P* < 0.001), age (*P* < 0.001), grade (*P* < 0.001), and radiation therapy (*P* = 0.0119) were highly correlated to tumor prognosis through univariate Cox regression (Figure 3A). Meanwhile, multivariate Cox regression analysis showed that TFRC expression was associated with overall survival independently (Figure 3B; *P* < 0.001). Subsequently, we used a nomogram model to predict the prognosis in patients with LGG based on Cox regression using the R package “regplot.” We found that TFRC expression contributes greatly to the outcome of the patient. Our internal validation of the nomogram showed a consistency index (C-index) of 0.819 (Figure 3C), indicating a relatively reliable predictive performance, the ROC curve showed that the area under curve (AUC) values at 1, 3, and 5 years were 0.852, 0.854, and 0.888, respectively (Figure 3D). The calibration plot for the 1-, 3-, and 5-year overall survival rate in LGG exhibited agreements between predictive survival probability and actual overall survival percentage (Figure 3E). In addition, DCA curves displayed a superior prognostic accuracy of OS and showed more clinical benefits (Figures 3F,G).

### DNA methylation and survival

**FIGURE 4:**
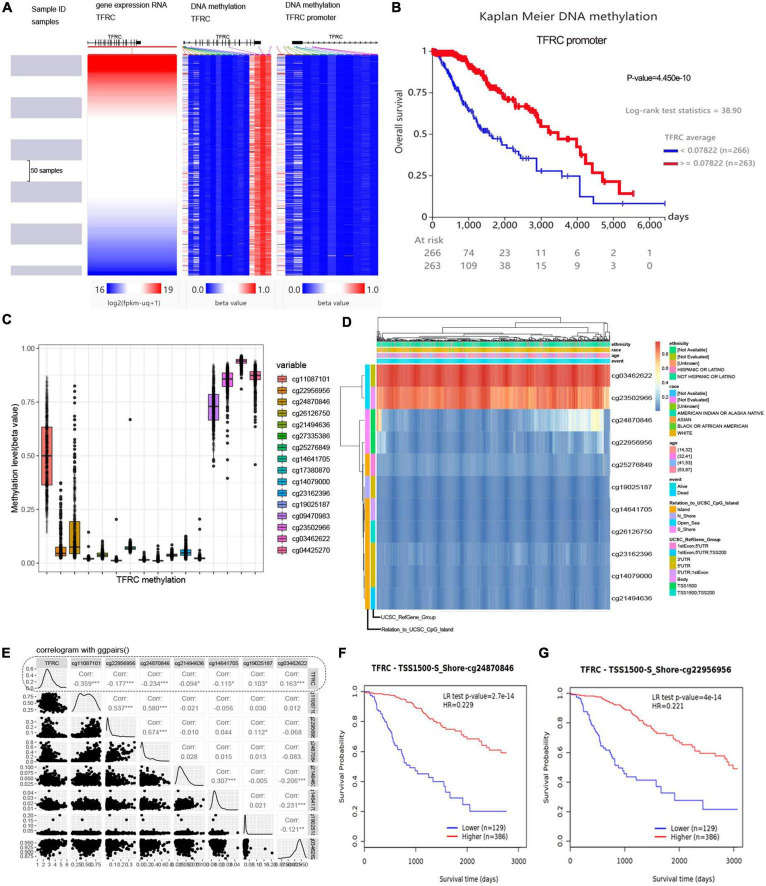
The DNA hypomethylation and gene expression of TFRC in LGG. **(A)** The UCSC Xena shows the gene expression and methylation level. **(B)** Survival analysis in TFRC promoter region. **(C,D)** Methylation levels of DNA sites in the presence of genomic regions and clinicopathological information. **(E)** Hypomethylation of the TFRC promoter region was negatively correlated with gene expression. **(F,G)** Kaplan–Meier survival curve in methylation of TFRC promoter region. *p < 0.05, ***p < 0.001.

The UCSC Xena database was employed to show the gene expression and methylation level of TFRC in LGG. The methylation status of the TFRC promoter in LGG showed hypomethylation throughout the TFRC gene coordinates region analysis (Figure 4A), and the promoter hypomethylation of DNA upstream of TFRC could lead to poor overall survival (Figure 4B). We showed methylation levels of 16 DNA sites in the presence of genomic regions of TFRC through the MethSurv tool (Figures 4C,D). Hypomethylation of the TFRC promoter region was negatively correlated with gene expression of TFRC, such as the cg22956956 and cg24870846 sites (Figure 4E). Meanwhile, the promoter hypomethylation in the cg22956956 and cg24870846 sites of TFRC predicts poor overall survival (Figures 4F,G, *p* < 0.01).

### Transferrin receptor protein 1 expression associated with tumor microenvironment and immune checkpoints in lower-grade glioma

**FIGURE 5:**
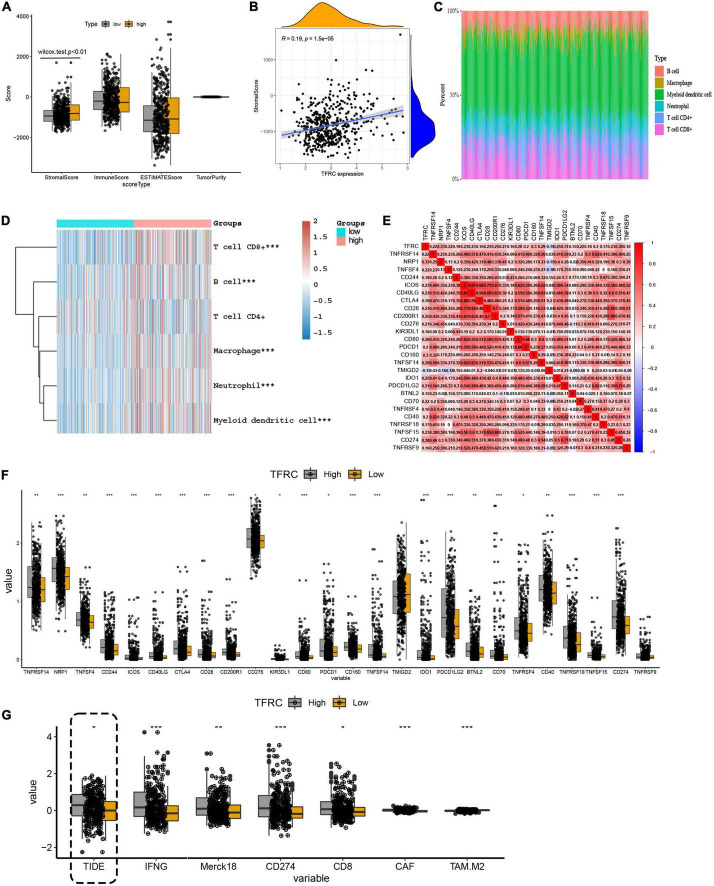
The influence of TFRC on tumor microenvironment, immune checkpoints, and ICB response in LGG. **(A,B)** Microenvironment scores. **(C)** Distribution of immune cells. **(D)** TFRC mRNA expression relationship with different immune cells in TCGA LGG tissue. **(E,F)** The influence of TFRC expression level on immune checkpoints. **(G)** The influence of TFRC expression level on the ICB response. *p < 0.05, **p < 0.01, ***p < 0.001.

For the tumor microenvironment, the ESTIMATE algorithm identified that TFRC expression was associated with the stromal score (*p* < 0.01), not with the immune score and tumor purity in LGG (Figures 5A,B). That is to say, with the increased stromal cell content, the expression of TFRC was increased. The distribution of immune cells was also analyzed (Figure 5C). The heatmap showed that high expression of TFRC was related to increased immune cells, such as CD8 + T cells, macrophage cells, and myeloid dendritic cells (*p* < 0.01) in LGG (Figure 5D). Meanwhile, we performed a simple analysis of immune checkpoints that were associated with immunotherapy (Wang et al., 2019). Some immune checkpoint-related molecules had a close correlation with TFRC expressions, such as PD-L1 (cd274) and CTLA4 (Figures 5E,F).

Then, we analyzed the immune checkpoint blockade (ICB) response to predict the immunotherapy effect in LGG. The TIDE algorithm showed that elevated expression of TFRC was related to high TIDE scores, suggesting tumor immune dysfunction and exclusion may occur (Figure 5G).

### Correlation of tumor mutational burden, ferroptosis, m6A, and transferrin receptor protein 1 expression in lower-grade glioma

**FIGURE 6:**
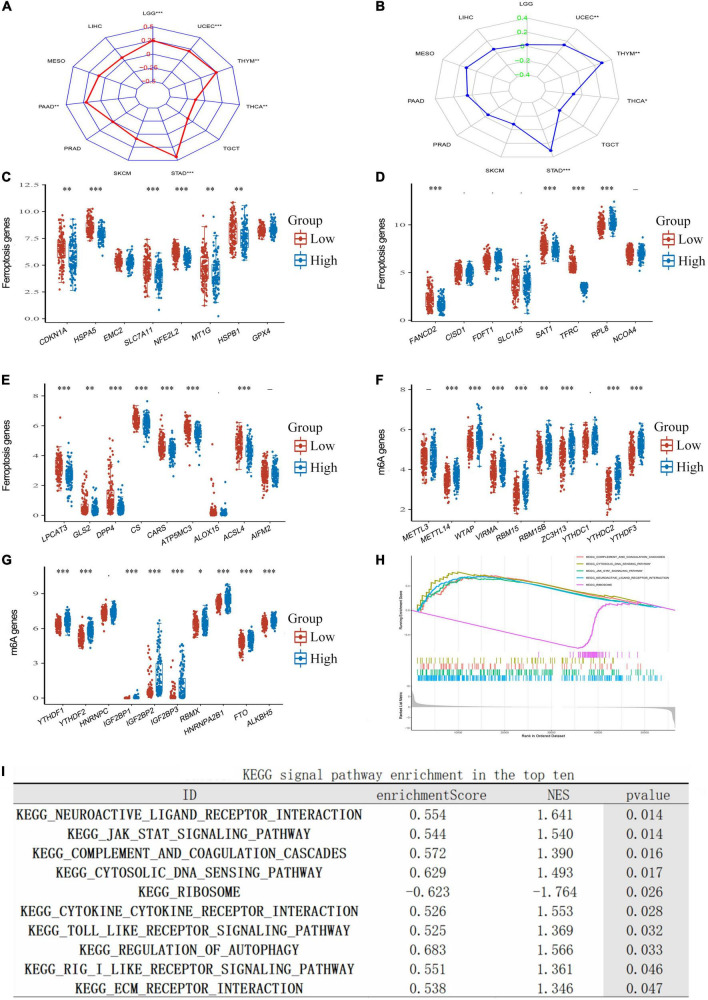
The correlation of TFRC expression and TMB, MSI, m6A methylation, ferroptosis, and enrichment analysis in LGG. **(A)** The correlation between TFRC levels and TMB. **(B)** The correlation of TFRC levels and MSI. **(C–E)** The correlation of TFRC levels and ferroptosis. **(F,G)** The correlation of TFRC levels and m6A methylation. **(H,I)** Enrichment plots from gene set enrichment analysis (GSEA). *p < 0.05, ^**^p < 0.01, ^***^p < 0.001.

Tumor mutational burden (TMB) is a promising immune-response biomarker and a potential spearhead in advancing targeted therapy trials (Choucair et al., 2020). We found that TFRC expression in LGG significantly influenced TMB (Figure 6A, *P* < 0.01); programmed death 1 (PD-1) blockade had clinical benefits in microsatellite-instability-high (MSI-H) or mismatch-repair-deficient (dMMR) tumors after the previous therapy (André et al., 2020). However, we found no significant correlation between MSI and TFRC expression in LGG (Figure 6B). TFRC has an obvious correlation with ferroptosis-related genes, such as HSPA1, SLC7A11, and NFE2L2 (Figures 6C–E). For N6-methyladenosine (m6A) RNA modification, we found that a variety of m6A modification-related genes had an obvious relation with TFRC expression, such as METTL4, WTAP, and VIRMA (Figures 6F,G).

### Gene set enrichment analysis, gene ontology, and Kyoto Encyclopedia of Genes and Genomes analysis

To explore the biological roles of TFRC in LGG, we performed GSEA to find the gene sets or tumor-associated pathways. Several immune-related pathways (such as complement and coagulation cascades, cytosolic DNA sensing pathway, the Janus kinase-signal transducer and activator of transcription pathway (JAK-STAT signaling pathway), and neuroactive ligand–receptor interaction) were also differentially enriched in the high TFRC expression group (Figures 6H,I).

**FIGURE 7:**
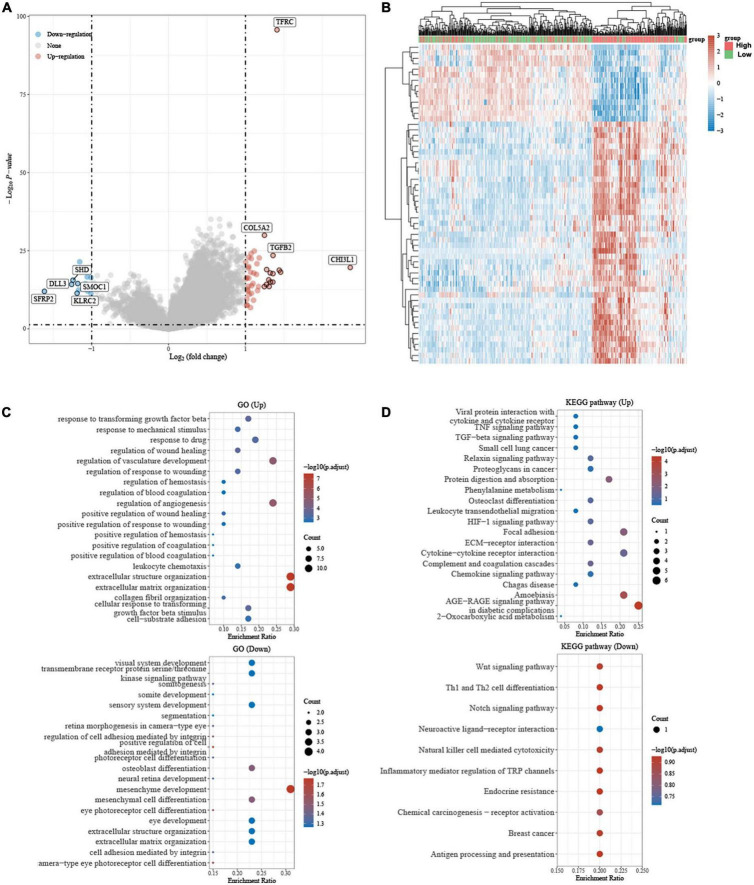
The differential gene expression and Gene ontology (GO), Kyoto Encyclopedia of Genes and Genomes (KEGG) Enrichment Analysis. **(A)** Volcano plot, red dots: upregulated genes; blue dots: downregulated genes. **(B)** Heat map: Different colors represent gene expression trends in different tissues. **(C,D)** GO and KEGG analysis showed that the biological functional enrichment, the circle’s size represents how many genes are differentially enriched, and the color represents its significance.

The volcano plot and heatmap showed that differential gene expression in different groups of patients with LGG based on the median value of TFRC (Figures 7A,B). To further confirm the potential function of TFRC, we employed gene ontology (GO) and Kyoto Encyclopedia of Genes and Genomes (KEGG) enrichment analysis, and the result also showed that tumorigenesis and immune-related pathways were associated with TFRC (Figures 7C,D).

### Experiment validation and single-cell analysis

**FIGURE 8:**
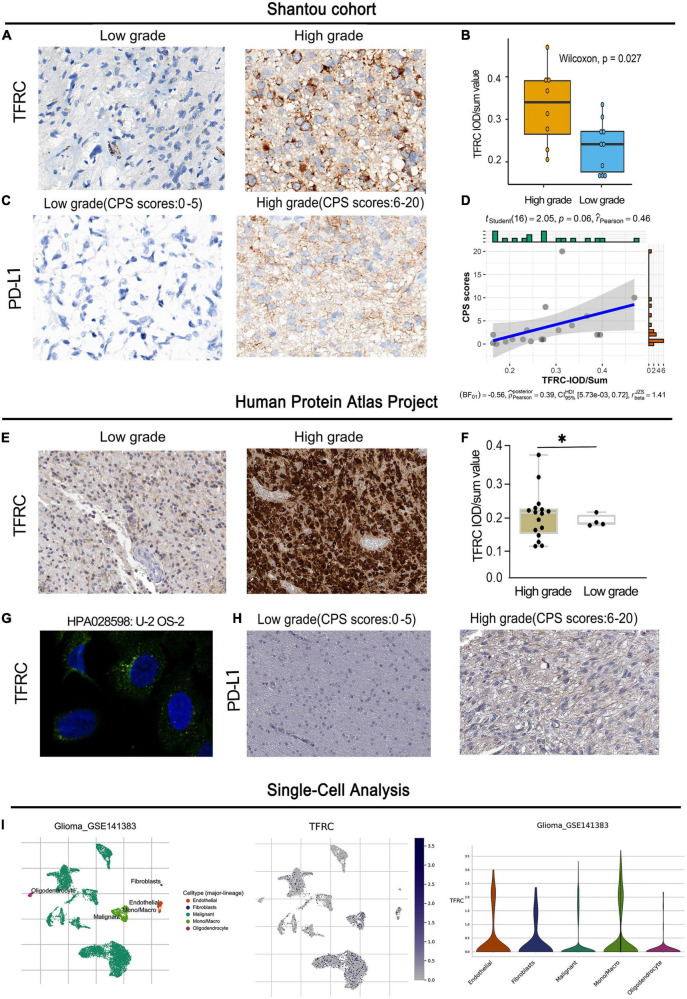
Immunohistochemistry and single-cell analysis of TFRC and PD-L1 expression in LGG. **(A–D)** Shantou project. **(E–H)** Human Protein Atlas project. **(I)** Single-cell analysis of TFRC. ^**^p < 0.01.

In the Shantou cohort, the result showed that the TFRC protein expression in the high-grade group of LGG was higher than those in the low-grade group, and the spatial distribution of TFRC was mainly in tumor cytoplasm and blood vessels by the immunohistochemical method (Figures 8A,B). According to the CPS score, the expression of PD-L1, there may have a certain correlation between PDL1 expression and TFRC (*R* = 0.46) but the *p*-value is 0.06 in LGG (Figures 8C,D).

We also obtained experimental data from the Human Protein Atlas Project. For 21 immunohistochemistry staining slides of glioma, we re-evaluated independently by two experienced pathologists. The result showed that the expression of TFRC in the high-grade group of LGG was higher than those in the low-grade group (Figures 8E,F). Studies using immunofluorescence and subcellular localization showed that TFRC proteins are primarily located in the cytoplasm (Figure 8G). According to the CPS score, the expression of PD-L1 in the high-grade group tended to be higher than those in the low-expression group in LGG (Figure 8H). These results suggest that TFRC has a certain correlation with prognosis and immunity.

For a single-cell analysis, we employed the TISCH2 tool to understand the TFRC expression comparison with different cell populations. The result showed that TFRC was mainly expressed in tumor cells, vascular endothelial cells, and macrophages (Figure 8I).

## Discussion

With the development of medicine, the diagnosis and treatment of the brain glioma (LGG) have been more and more in-depth and clear. However, there are still many unknowns about the molecular mechanism of its occurrence, development, and prognosis.

Ferroptosis has been demonstrated to inhibit tumor growth (Liu et al., 2020). However, some reports found that the ferroptosis-related molecular TFRC can induce the activation of ferroptosis in neuroblastoma (NB) (Yuxiong et al., 2021). In addition, we found that TFRC has a high expression level in several cancers, including LGG. Patients with LGG and high TFRC expression levels have a poor prognosis. Therefore, we speculated that TFRC might have a dual role in regulating tumor progress. Alternatively, the increased expression pattern of TFRC may be an adaptive compensatory response for LGG.

To understand the correlation between TFRC and tumor clinicopathological factors, we conducted a correlation analysis in LGG. According to the five editions of the WHO classification of the central nervous system, Isocitrate dehydrogenase (IDH) mutations are related to better survival outcomes for patients with glioma (Louis et al., 2021). Our study illustrated that the TFRC positively correlates with tumor progress factors, such as IDH mutation status and clinical tumor grade. Thus, the results demonstrated that TFRC plays a certain role in influencing tumor progression.

To further characterize the effect of TFRC on tumor prognosis, we performed univariate and multivariate analyses using the Cox-proportional hazards model. As a result, TFRC molecules were identified as significant prognostic factors in LGG. Furthermore, through the nomogram prognosis model (Wang et al., 2018), we found that the TFRC expression contributes greatly to the patient’s outcome.

For gene regulation, it is generally believed that promoter hypomethylation is associated with high gene expression (Razin and Kantor, 2005). Multiple omics analyses were used to clarify the upstream mechanisms affecting the TFRC expression. We found that DNA methylation has a conspicuous influence on the TFRC expression and the overall survival of LGG. Therefore, we hypothesized that DNA methylation could influence survival by regulating the TFRC expression. However, its detailed mechanism should be further explored.

The interaction between ferroptosis and immunity has been a topic of substantial interest since its discovery in 2012 (Shi et al., 2021). However, it is not clear whether the ferroptosis-related molecule TFRC affects tumor immunity in LGG. In this study, our research showed that the high expression of TFRC has been associated with tumor-infiltrating lymphocyte (TIL) levels (CD8 + T cell, Macrophage cell) and immune checkpoints, suggesting a novel immunotherapy target.

Tumor mutational burden (TMB) and microsatellite instability (MSI) are genomic biomarkers used to identify patients who are likely to benefit from immune checkpoint inhibitors (Jardim et al., 2021; Palmeri et al., 2022). Therefore, we carried out those primary analyses to clarify the relationship between TFRC and tumor immunotherapy. Our results showed that the TFRC expression was positively correlated with TMB in LGG and had no obvious correlation with MSI.

N6-methyladenosine (m6A) methylation plays an important role in the genesis and development of various tumors (Liuer et al., 2019; Yi et al., 2020). Our study found that TFRC expression is highly correlated with m6A writer, m6A eraser, and m6A reader-related genes. Furthermore, we found that all m6A-related genes in LGG tissues were positively correlated with TFRC, and it was speculated that TFRC expression was affected by the m6A modification in the RNA transcription level in LGG.

To further explore the molecular function of TFRC in LGG, we conducted a GSEA, GO, and KEGG analysis, and we found that autophagy, ECM–receptor interaction signaling pathways, and complement and coagulation cascades were differentially enriched in the TFRC expression phenotype. From a molecular mechanism perspective, this result also demonstrated that TFRC might promote tumor progression and is closely related to tumor immunity.

At last, we demonstrated the correlation of TFRC with prognosis and immune checkpoints (PD-L1) by experimental analysis. This is consistent with the results of previous bioinformatics methods. Moreover, the single-cell analysis showed TFRC expression comparison in different cell populations, especially in vascular endothelial cells and macrophages. This helps us understand the relationship between TFRC and metastasis and the cell-cell interaction of the immune microenvironment.

This study is based on bioinformatics and preliminary experimental methods to investigate the role of TFRC. Therefore, this study has some limitations.

In conclusion, our study showed that the TFRC could lead to a poor prognosis in LGG. It may also be an effective target for immunotherapy for patients with LGG.

## Data availability statement

The original contributions presented in the study are included in the article/Supplementary material, further inquiries can be directed to the corresponding author.

## Ethics statement

This study was approved by the Ethics Committee of The First Affiliated Hospital of Shantou University Medical College (Shantou, Guangdong Province, China).

## Author contributions

HW and LH designed the study. HH carried out data acquisition and analysis. HH and HW wrote the manuscript and contributed to preparing and making figures and tables. JH provided guidance in clinicopathological diagnosis. CW, YD, RL, ZC, and QQ participated in the immunohistochemical experiments. All authors read and approved the final manuscript.

## Footnotes

1
http://xena.ucsc.edu/

2
http://www.cgga.org.cn

3
https://www.oncomine.org/resource/main.html

4
https://www.proteinatlas.org
