# Supplementary figures and images for: Identification and validation of transferrin receptor protein 1 for predicting prognosis and immune infiltration in lower grade glioma
Source: Front Mol Neurosci. 2022 Nov 22;15:972308. doi: 10.3389/fnmol.2022.972308 (PMC9723399; doi:10.3389/fnmol.2022.972308)

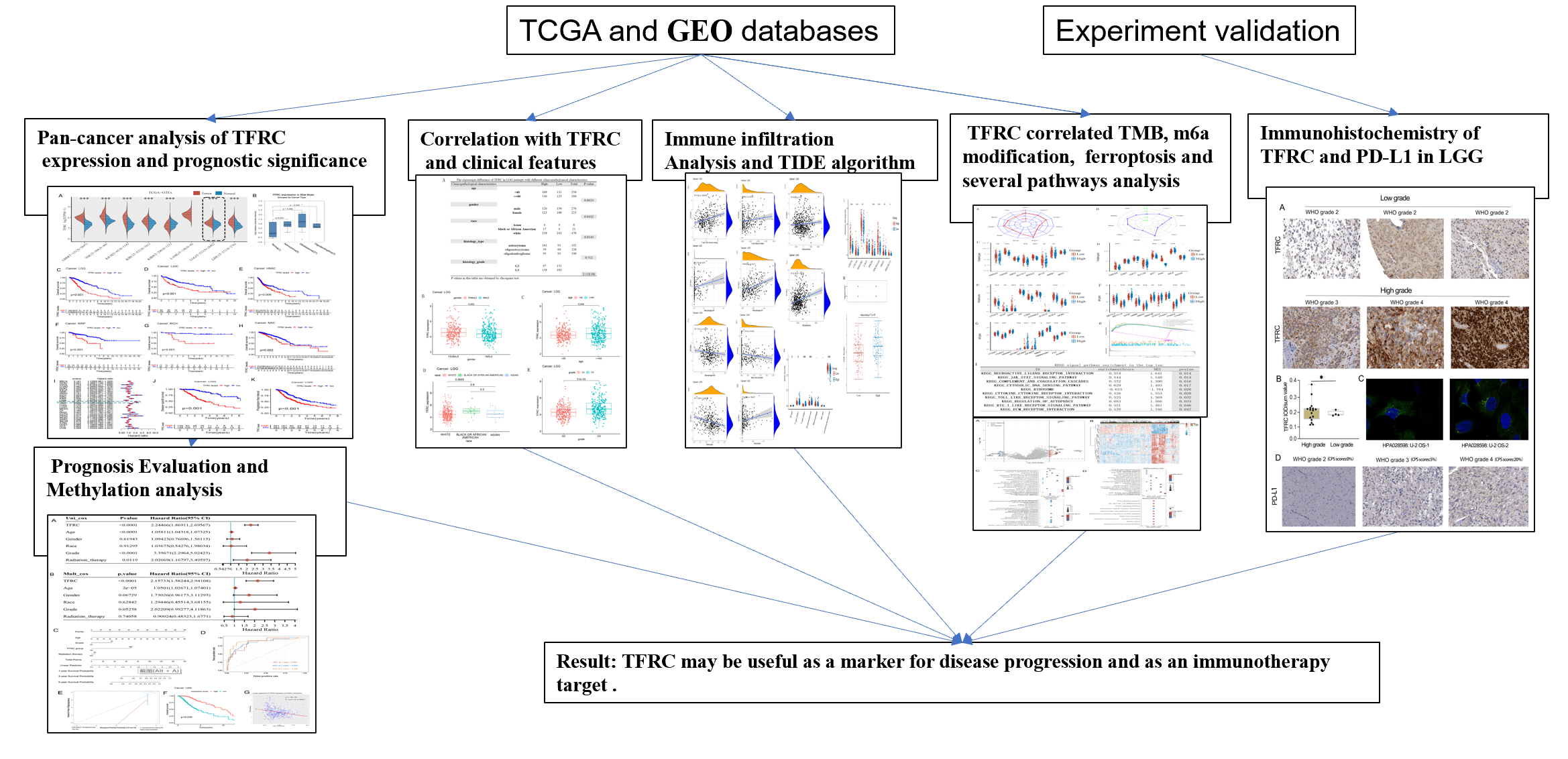

Supplement: Supplementary file 1 [file Image_1.tif]

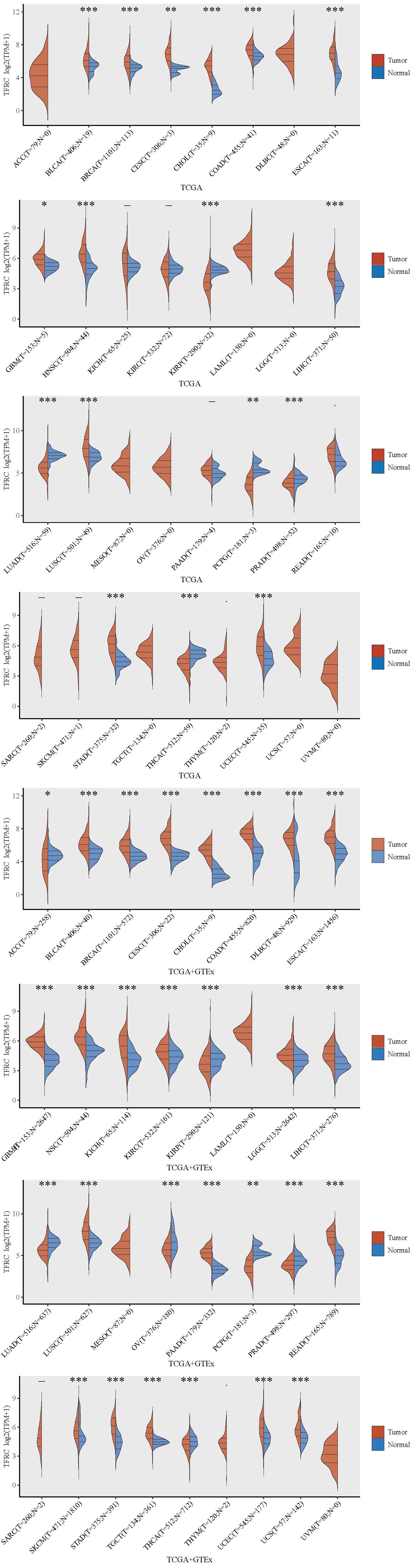

Supplement: Supplementary file 2 [file Image_2.tiff]
